# Gene expression profiling of the hyperplastic growth zones of the late trout embryo myotome using laser capture microdissection and microarray analysis

**DOI:** 10.1186/1471-2164-14-173

**Published:** 2013-03-14

**Authors:** Pierre-Yves Rescan, Jerome Montfort, Alain Fautrel, Cécile Rallière, Veronique Lebret

**Affiliations:** 1 INRA, UR1037 LPGP Fish Physiology and Genomics, Rennes F-35000, France; 2 Histopathology Platform H2P2, Biosit/Biogenouest, Rennes, France

**Keywords:** Myogenesis, Muscle growth, Muscle hyperplasia, Gene expression, Transcriptome, Laser capture microdissection, Teleost

## Abstract

**Background:**

A unique feature of fish is that new muscle fibres continue to be produced throughout much of the life cycle; a process termed muscle hyperplasia. In trout, this process begins in the late embryo stage and occurs in both a discrete, continuous layer at the surface of the primary myotome (stratified hyperplasia) and between existing muscle fibres throughout the myotome (mosaic hyperplasia). In post-larval stages, muscle hyperplasia is only of the mosaic type and persists until 40% of the maximum body length is reached. To characterise the genetic basis of myotube neoformation in trout, we combined laser capture microdissection and microarray analysis to compare the transcriptome of hyperplastic regions of the late embryo myotome with that of adult myotomal muscle, which displays only limited hyperplasia.

**Results:**

Gene expression was analysed using Agilent trout oligo microarrays. Our analysis identified more than 6800 transcripts that were significantly up-regulated in the superficial hyperplastic zones of the late embryonic myotome compared to adult myotomal muscle. In addition to Pax3, Pax7 and the fundamental myogenic basic helix-loop-helix regulators, we identified a large set of up-regulated transcriptional factors, including Myc paralogs, members of Hes family and many homeobox-containing transcriptional regulators. Other cell-autonomous regulators overexpressed in hyperplastic zones included a large set of cell surface proteins belonging to the Ig superfamily. Among the secreted molecules found to be overexpressed in hyperplastic areas, we noted growth factors as well as signalling molecules. A novel finding in our study is that many genes that regulate planar cell polarity (PCP) were overexpressed in superficial hyperplastic zones, suggesting that the PCP pathway is involved in the oriented elongation of the neofibres.

**Conclusion:**

The results obtained in this study provide a valuable resource for further analysis of novel genes potentially involved in hyperplastic muscle growth in fish. Ultimately, this study could yield insights into particular genes, pathways or cellular processes that may stimulate muscle regeneration in other vertebrates.

## Background

In the myotome of teleost fish, new muscle fibres continue to be produced far into adulthood, whereas in mammals postnatal growth depends only on the hypertrophy of muscle fibres that are formed during embryonic development [1]. The post-embryonic formation of muscle fibres in fish generally occurs in two successive phases [2]. In the first phase, which takes place in the late embryo stage and/or in larvae, new fibres are formed at the surface of the primary myotome. This regionalised phase of myogenesis, termed stratified hyperplasia, results from the differentiation of myogenic precursor cells that derive from the dermomyotome-like epithelium that surrounds the myotome [3-6]. In the second phase of neomyogenesis, termed mosaic hyperplasia, new muscle fibres are formed on the surface of existing muscle fibres throughout the entire myotome, producing the typical mosaic appearance observed in a muscle cross section. Mosaic hyperplasia is responsible for the robust increase in muscle mass in larvae and in juveniles [7]. Myogenic precursor cells that underlie the basal lamina of mature muscle fibres power mosaic hyperplasia [8]. These resident quiescent cells, which are the equivalent of mammalian satellite cells, express FoxK1 protein [9], a member of the forkhead/winged helix family of transcription factors and one of the few known markers of quiescent satellite cells in mammalian muscle [10]. Although it has not been formally demonstrated, it is likely that these satellite cells in fish also originate from the dermomyotome [3]. In most teleost species, the onset of mosaic hyperplasia follows stratified hyperplasia and begins only in the advanced larval stages [2]. In contrast, in trout, mosaic hyperplasia and stratified hyperplasia occur simultaneously, immediately following the differentiation of the embryonic myotome [11]. This mode of growth likely accounts for the intense embryonic body growth observed in salmonids [11]. Little is known about the genetic mechanisms regulating the formation of new myofibres in fish, primarily as a result of the difficulty of accessing the zones of myotube neoformation. In this study we have combined laser capture microdissection [12] with microarray analysis to compare the transcriptome of hyperplastic subdomains of the late embryo myotome with that of adult myotomal muscle, which displays only limited muscle hyperplasia.

## Methods

### Ethics Statement

This work used early trout embryos. All experiments performed in this study followed the recommendations of the “Comité National de Reflexion Ethique sur l’Experimentation Animale” of the Ministry of Higher Education and Research and were approved by Local Animal Care and Use Committee (approval n° 7I12).

### Animals and sampling

All experiments were performed using rainbow trout *Oncorhynchus mykiss* (Walbaum). Laser capture microdissection of myotome subdomains was carried out on 19 day-old prehatching trout embryos. RNA extraction of adult myotomal muscle was carried out using three distinct animals from a mixed-sex trout population and weighing approximately 500 grams. The trout were rapidly anaesthetised with phenoxyethanol (Sigma; 4 ml/10 litres fresh water) before sacrificing. A transverse slice of fast muscle situated just beneath the dorsal fin was then sampled and was stored at −80°C prior to RNA extraction using using TRIzol reagent (Invitrogen, Carlsbad, CA).

### Selective isolation of superficial and deep domains of the myotome of the late embryo by Laser Capture Microscopy (LCM)

Superficial growth zones under the slow muscle layer and subjacent primary myotome-derived muscle mass were separately isolated using laser capture microdissection. For this purpose, late trout embryos were frozen in liquid nitrogen-cooled isopentane. Ten-micron- thick transverse frozen sections were cut using a cryostat (Leica, Milton Keynes, UK), mounted onto uncoated glass slides, fixed at −20°C in 70% ethanol for 1 min, washed briefly in 70% ethanol and sequentially dehydrated in 100% ethanol and xylene. The sections were then microdissected using a Veritas Laser Capture Microdissection system (LCM) (Arcturus). The infrared laser was used with the following parameters: spot diameter, 20 μm; pulse duration, 3500 ms; power, 90 mW. All areas were selected and collected within 30 min of the preparation of the slide. A total of 20–30 laser-captured area obtained from 2–3 late embryos were pooled for each RNA extraction. Total RNA was isolated using the PicoPureRNA isolation kit (Arcturus) and had an RNA integrity number of 7.5 (Agilent).

### Microarray slides

Microarray experiments were performed using an Agilent-based microarray platform with 8 × 60 K probes per slide (GEO platform record: GPL15840). This platform, which is based on a rainbow trout resource designed by Salem *et al*. [13], has been enriched with oligonucleotides designed using recent NGS data from trout [14]. The microarray gene annotations were reanalysed by Sigenae (Institut National de la Recherche Agronomique, Toulouse, France). Microarray data sets have been submitted to the GEO-NCBI with the accession number GSE40410.

### RNA labelling and hybridisation

RNA from (i) four distinct pools of laser-captured superficial area of late trout embryo myotome, (ii) three distinct pools of laser-captured deep area of late trout myotome and (iii) three distinct adult fast muscles were used for labelling and hybridisation. RNA samples were Cy3-labelled according to the manufacturer’s instructions (Agilent). Briefly, RNA was first reverse transcribed, using a polyDT-T7 primer, Cy3 was incorporated by a T7 polymerase-mediated transcription and excess dye was removed using an RNeasy kit (Quiagen). The level of dye incorporation was evaluated using a spectrophotometer (Nanodrop ND1000, LabTech). Labelled RNA was then fragmented in the appropriate buffer (Agilent) for 30 minutes at 60°C before dilution (v/v) in hybridisation buffer. Hybridisations were performed in a microarray hybridisation Oven (Agilent) overnight at 65°C, using two Agilent 8 × 60 K high-density oligonucleotide microarray slides. Following hybridisation, the slides were rinsed in gene expression wash buffers 1 and 2 (Agilent).

### Data acquisition and analysis

Hybridised slides were scanned at a 3-μm resolution using an Agilent G2505 microscanner. Data were extracted using the standard procedures contained in the Agilent Feature Extraction (FE) software version 10.7. Arrays were normalised using GeneSpring software. A *t*-test (p < 0.01) and an average fold change of >3 were used as the criteria for defining genes as differentially expressed between hyperplastic areas of the late embryonic myotome and adult myotomal muscle. For clustering analysis, data were log transformed, median-centred and an average linkage clustering was carried out using CLUSTER software. The results were visualised using TREEVIEW [15]. Biological functions and pathways were generated and analysed using Ingenuity Pathway Analysis software (IPA, Ingenuity Systems, CA).

### In situ hybridisation

Recombinant bacterial clones were obtained from the CRB GADIE resource centre (Jouy-en-Josas, France) or the USDA (Washington D.C., USA). Plasmid were extracted and cDNA inserts were amplified by PCR using vector-specific primers. PCR products were purified and used as templates for digoxigenin (DIG)-labeled probe synthesis using the Riboprobe Combination system - T3/T7 RNA polymerase (Promega).

In situ hybridisation experiments were performed in 17 day-old trout embryos. Briefly trout embryos were dechorionated with fine forceps and were fixed overnight at 4°C with paraformaldehyde in phosphate buffered saline (PBS). Specimens were dehydrated and stored in methanol at −20°C. After rehydration in graded methanol-PBS, embryos were processed according to established automated procedures [16] with minor modifications.

## Results

### Muscle hyperplasia in the late trout embryo

**Figure 1:**
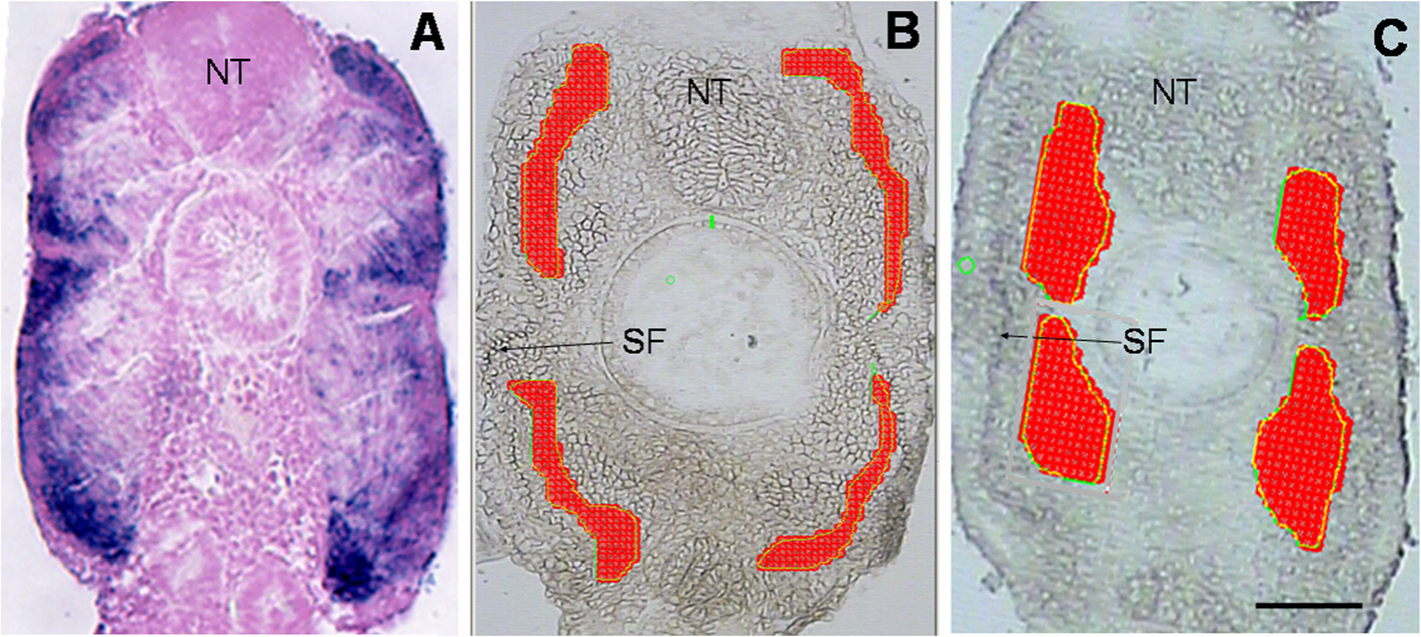
**(A) Expression of myogenin in 19-day-old trout embryos.** Transverse section: labelling can be observed at the periphery of the myotome, and, in a scattered pattern, throughout the deep primary myotome-derived muscle mass. (**B**-**C**) Representative laser capture microdissection of peripheral (**B**) and deep domains (**C**) of the myotome of 19-day-old trout embryos. The areas in red correspond to laser-captured surfaces. SF: slow fibres. Horizontal line, 75 μm.

The zones of myotube neoformation in the trout late embryonic myotome were visualised by in situ hybridisation of the transcript encoding myogenin, a transcriptional activator associated with the differentiation of myogenic cells. Transverse sections from 19-day-old trout embryos revealed that myogenin was strongly expressed in a discrete layer at the periphery of the myotome, including the dorsal and ventral extremes, and, in a scattered pattern, throughout the deep primary myotome-derived muscle mass (Figure 1A). These observations indicate that the predominant mode of hyperplasia in trout late embryos is stratified and occurs at the periphery of the expanding myotome. However, a deeper, sparse distribution of myogenin labelling indicates the concomitant mosaic differentiation of new fibres in the primary myotome-derived muscle mass. This pattern is in agreement with that previously reported in brown trout [11].

### Gene expression profiling overview

**Figure 2:**
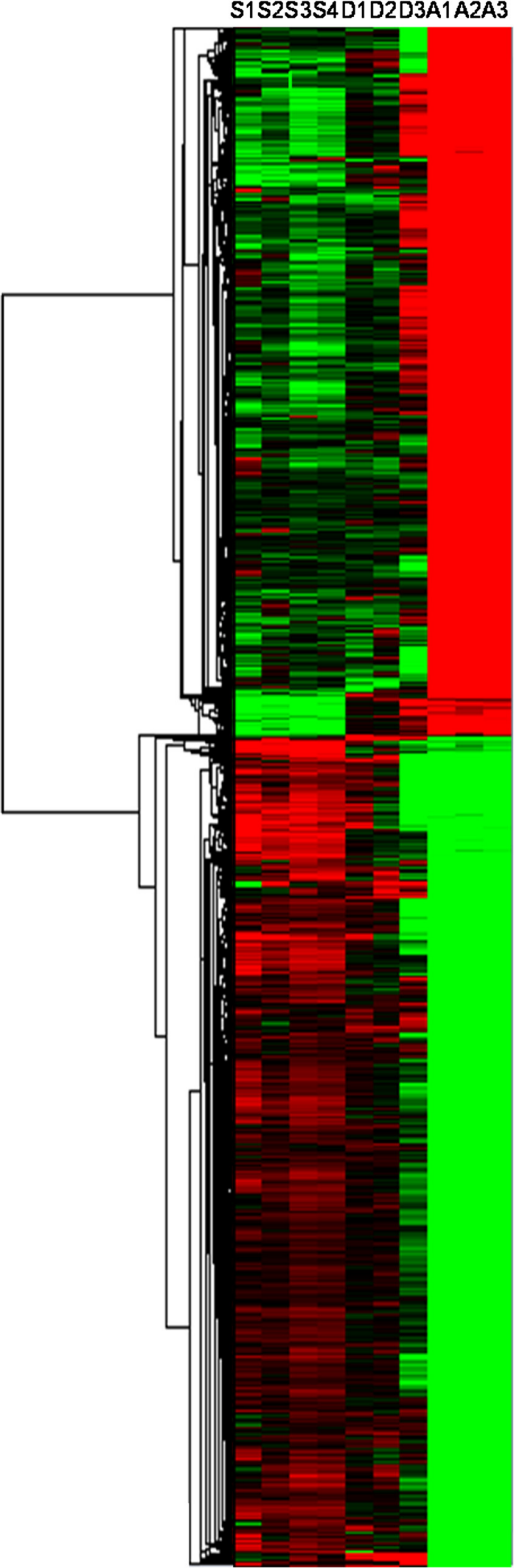
**Heat map of the hierarchical clustering of genes differentially expressed (minimum fold change >3; *P- *value > 0.01) between laser-captured superficial hyperplastic areas of the late embryo myotome and adult myotomal muscle.** The horizontal dendrogram represents the correlation distances between gene expression levels. Each row represents the expression of a single gene and each column represents a single sample as follows: columns 1–4 (S1-S4), superficial hyperplastic zones of the late embryonic myotome; columns 5–7 (D1-D3), deep zones of the late embryonic myotome; columns 8–10 (A1-A3), adult muscle. Expression levels are represented by a colour tag, with red representing high levels of expression and green representing low levels of expression.

**Figure 3:**
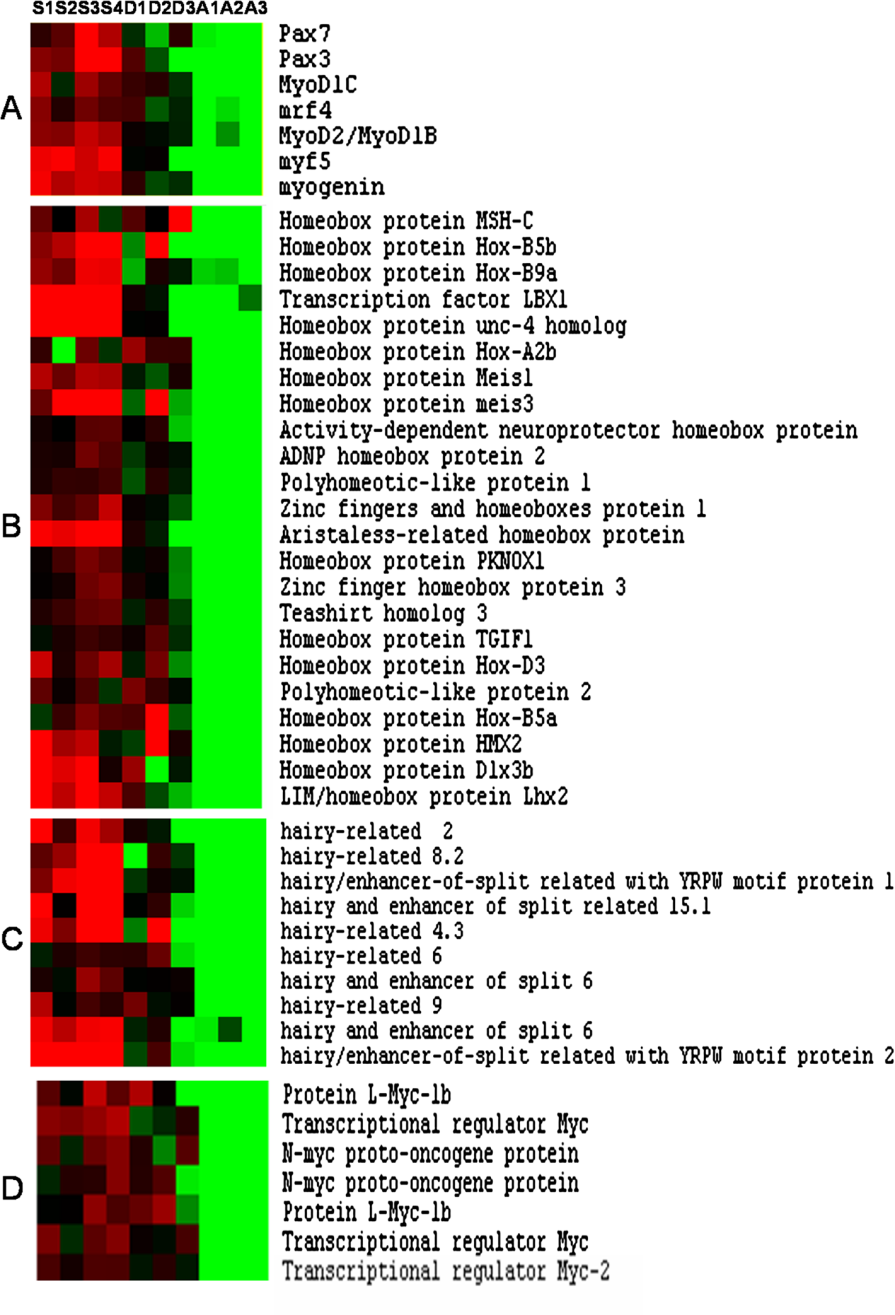
**Supervised clustering of transcriptional regulators overexpressed in hyperplastic areas.** (**A**) canonical myogenic markers and bHLH regulators, (**B**) homeobox containing transcriptional factors, (**C**) members of the Hairy/enhancer of split (Hes) family and (**D**) Myc paralogs. Columns are as in Figure 2. Few genes are present as multiple copies resulting from paralogue retention following whole genome duplication (WGD) events that occurred at the base of the actinopterigyans or specific to the salmonid lineage.

Superficial growth zones located beneath the slow muscle and deep primary myotome-derived muscle mass were separately laser-captured from transverse sections of 19-day-old trout embryos (Figure 1B and C). Four distinct pools of laser-captured superficial domains, three distinct pools of laser-captured deep domains and three distinct adult fast muscles were used for microarray experiments. An average fold change of >3 and P <0.01 were used as the criteria for defining genes as differentially expressed between laser-captured growth zones located at the periphery of the late embryo myotome and adult muscle. The supervised clustering of the differentially expressed genes is shown in Figure 2 and is available online as a browseable file [17]. Overall, 6828 genes were found to be up-regulated and 5841were found to be down-regulated in superficial hyperplastic areas of the late embryo myotome compared to adult muscle. Consistent with the hyperplastic growth pattern in trout [11], hyperplasia-correlated genes exhibit intermediate expression in deep primary myotome-derived muscle mass compared to the expression observed in superficial hyperplastic areas and in adult muscle. This is exemplified in the expression of Myogenic regulatory factors including myogenin (Figure 3A). Ingenuity pathways analysis software (IPA) was used to determine significant biological functions associated with hyperplastic areas. A total of 5497 genes were identified as eligible by IPA and were used for functional analysis. As shown in Additional file 1, top networks and major molecular and cellular functions associated with muscle hyperplasia-correlated genes were related to RNA processing, protein synthesis, DNA replication recombination and repair and cell cycle. “Embryonic development”, “Organismal development”, “Tissue development”, and “Tissue morphology”, were the top significant categories in “physiological development and system function” (Additional file 1). The category “Embryonic development” included the functional annotation “myogenesis of embryonic cell lines” while the category “Tissue development” included the functional annotation “morphogenesis of muscle” (not shown).

### Identifying genes associated with myotube formation

Muscle fibre hyperplasia involves the proliferation, fusion and differentiation of myogenic cells. These events are regulated by an interplay of intrinsic factors and extrinsic signals. Therefore, to further identify candidate genes of specific relevance in the regulation of muscle hyperplasia, we focused on transcripts encoding cell-autonomous (intrinsic) factors such as transcriptional regulators and membrane associated proteins, and on transcripts encoding extrinsic factors such as secreted factors, including growth factors and signalling molecules.

### Transcriptional regulators: DNA-binding transcriptional regulators

More than 100 DNA-binding transcriptional regulators were found to be up-regulated in the superficial hyperplastic areas of the late embryonic myotome when compared to adult fast muscle (Additional file 2). Among these factors were well known regulators of muscle-specific transcription such as the paired-box transcription factors, Pax3 and Pax7, which mark immature myogenic cells, and the myogenic bHLH transcription factors such as MyoD (Myod1b and MyoD1c), Myf5, mrf4 and myogenin, which act downstream of the pax3/7 genes to trigger myogenic differentiation (Figure 3A). In addition to these canonical myogenic regulators, we found a large set of genes encoding homeobox motif-containing transcriptional regulators such as members of the meis family (meis1 and meis3), activity-dependent neuroprotector homeobox protein, Lbx1 and *ARX* (Aristaless-related homeobox gene) (Figure 3B). Several genes found to be up-regulated in our analysis encode transcriptional regulators of the Sox family, such as Sox5, sox8 and sox11. Also were found the winged helix factor Foxc1 as well as Tbx2 and Tbx3 which are members of the T-box DNA binding-containing protein family. Among the transcriptional regulators with zinc finger motifs, we identified Gli2 and snail2, as well as Zic2 and Zic4. A salient feature of the hyperplastic zones was the strong enrichment of genes encoding members of Hairy/enhancer of split (Hes) family proteins such as hairy and enhancer of split 6, as well as Hes-related transcriptional factors including hairy enhancer of split with YRPW motif protein 1 (Hey1) and 2 (Hey2) (Figure 3C). Seven distinct c-Myc paralogs were up-regulated in hyperplastic areas (Figure 3D) along with their associated factor Max and the upstream transcriptional regulators the FUSE binding proteins FUBP1, FUBP2 and FUBP3. Finally, we observed an enrichment for members of the AP-2 family (alpha, alpha-2, epsilon and sigma) in hyperplastic zones.

### Transcriptional regulators: epigenetic factors

**Figure 4:**
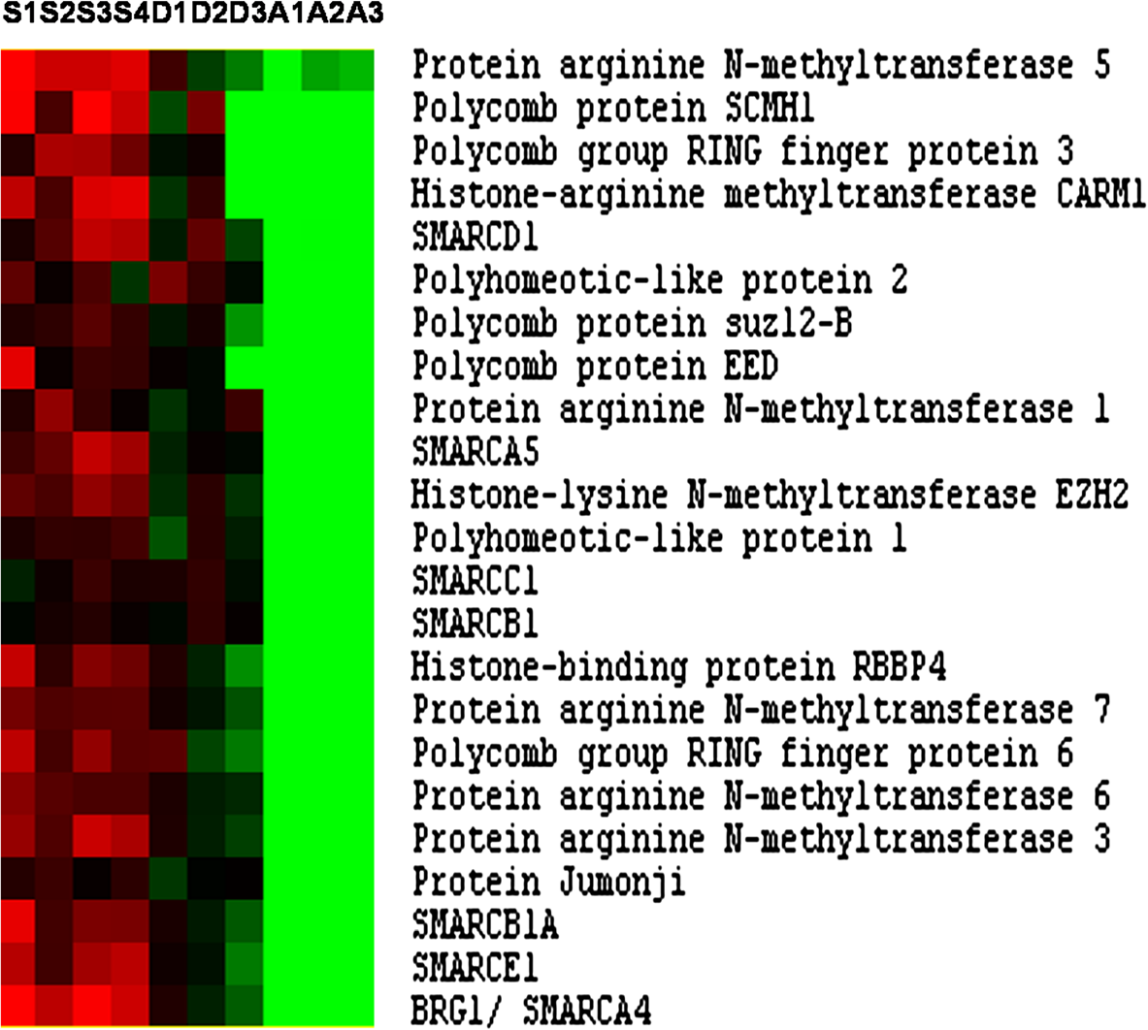
**Supervised clustering of epigenetic transcriptional regulators overexpressed in hyperplastic areas.** Columns are as in Figure 2.

A salient feature of the expression profiling of the superficial hyperplastic areas of the late embryonic myotome, when compared to adult fast muscle, was the strong enrichment for genes encoding epigenetic transcriptional regulators. Among these regulators were proteins belonging to the Polycomb groups (Figure 4), including Polycomb protein SCMH1, Polycomb-group ring finger proteins 3 and 6, and polyhomeotic-like proteins 1 and 2, all of which are components of the multiprotein PRC1-like complex [18]. In our expression profiling we also identified the histone-lysine N-methyltransferase EZH2, RBBP4, polycomb protein suz12-B, polycomb protein EED and Jarid2 (protein Jumonji) all of which associate with the PCR2 complex [18]. In addition, several histone modifying enzymes of the protein arginine methyltransferases (PRMT) families, such as PRMT1, PMRT3, PRMT4/CARM1, PRTM5, PRTM6 and PRMT7 were found to be overexpressed in germinal zones (Figure 4). Another group of up-regulated epigenetic regulators included several SWI/SNF chromatin-remodelling enzymes (Smarcd1, Smarce1, Smarcb1A, smarca5, smarcad1, Smarcab1, Smarcc1 and Smarca4/BRG1).

### Membrane-associated proteins

**Figure 5:**
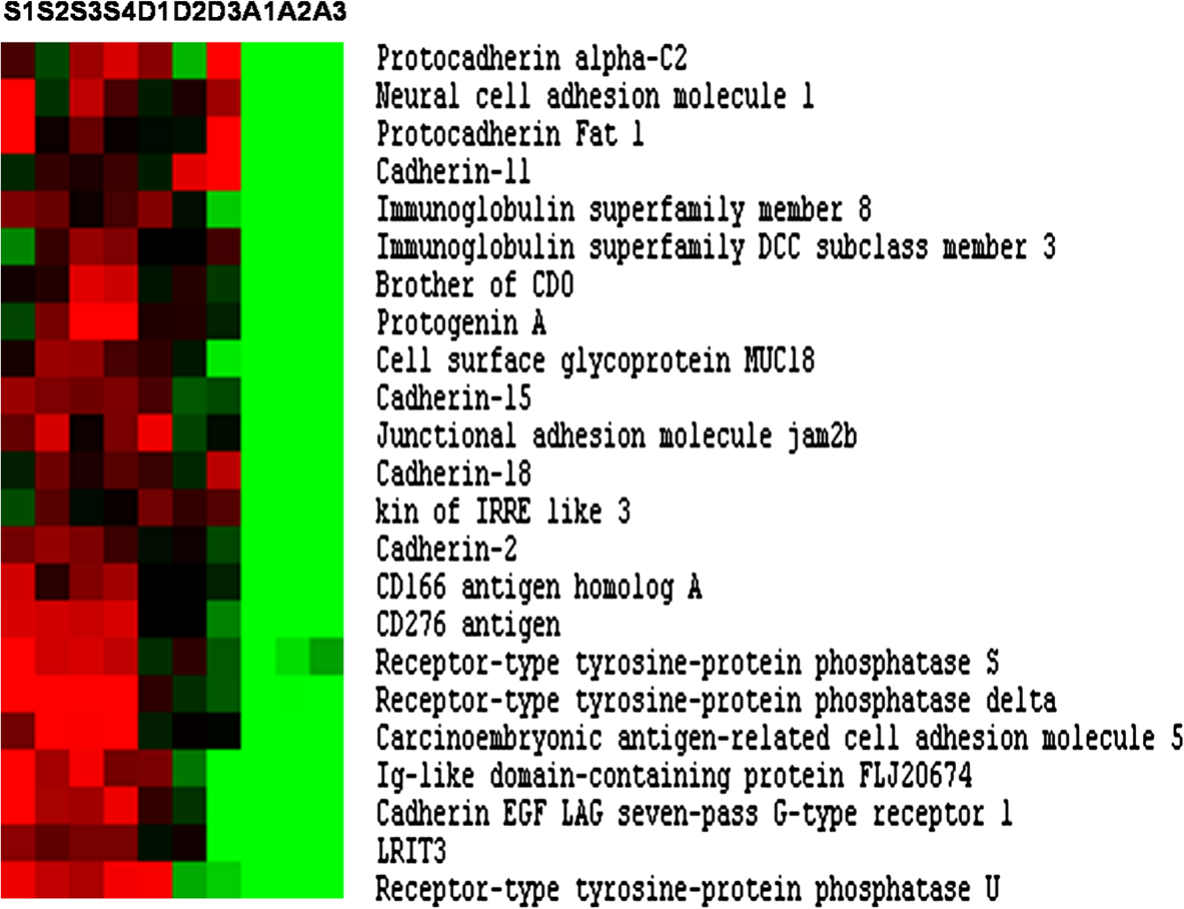
**Supervised clustering of immunoglobulin (Ig) domain-containing membrane receptors overexpressed in hyperplastic areas.** Columns are as in Figure 2.

More than 130 membrane protein-encoding genes were found to be overexpressed in superficial hyperplastic zones of the late embryo myotome compared to adult fast muscle (Additional file 3). Among these genes we observed a large set of immunoglobulin superfamily cell surface proteins, including the promyogenic cell surface receptors N-CAM, M-cadherin (cadherin 15), N-cadherin (cadherin 2), Kin of Irre 3, brother of CDO (BOC) and protogenin (Figure 5). Other transmembrane proteins containing Ig domain were also identified, including jam 2b and the receptor-linked protein tyrosine phosphatases U, sigma and delta (Figure 5). Transmembrane proteins overexpressed in hyperplastic zones also included Adam 17 and Adamts 18, which are members of the disintegrin and metalloprotease family. In addition, cleft lip and palate transmembrane protein 1, trophoblast glycoprotein, several tetraspanins, receptor binding cancer antigen expressed on SiSo cells /RCAS1, BMP receptor type IB, tissue factor/CD142, integrin alpha 9, the planar cell polarity effectors Vang-like protein 1 and 2, multiple ligand binding receptors such as fibroblast growth factor receptor 4, Ephrin type A receptor 2 and Ephrin type B receptor 3, TNF receptor member 19 and Frizzled receptors (1, 3, 7, 8 and 10) were all up-regulated in the microarray.

### Signalling environment components and other secreted factors

**Figure 6:**
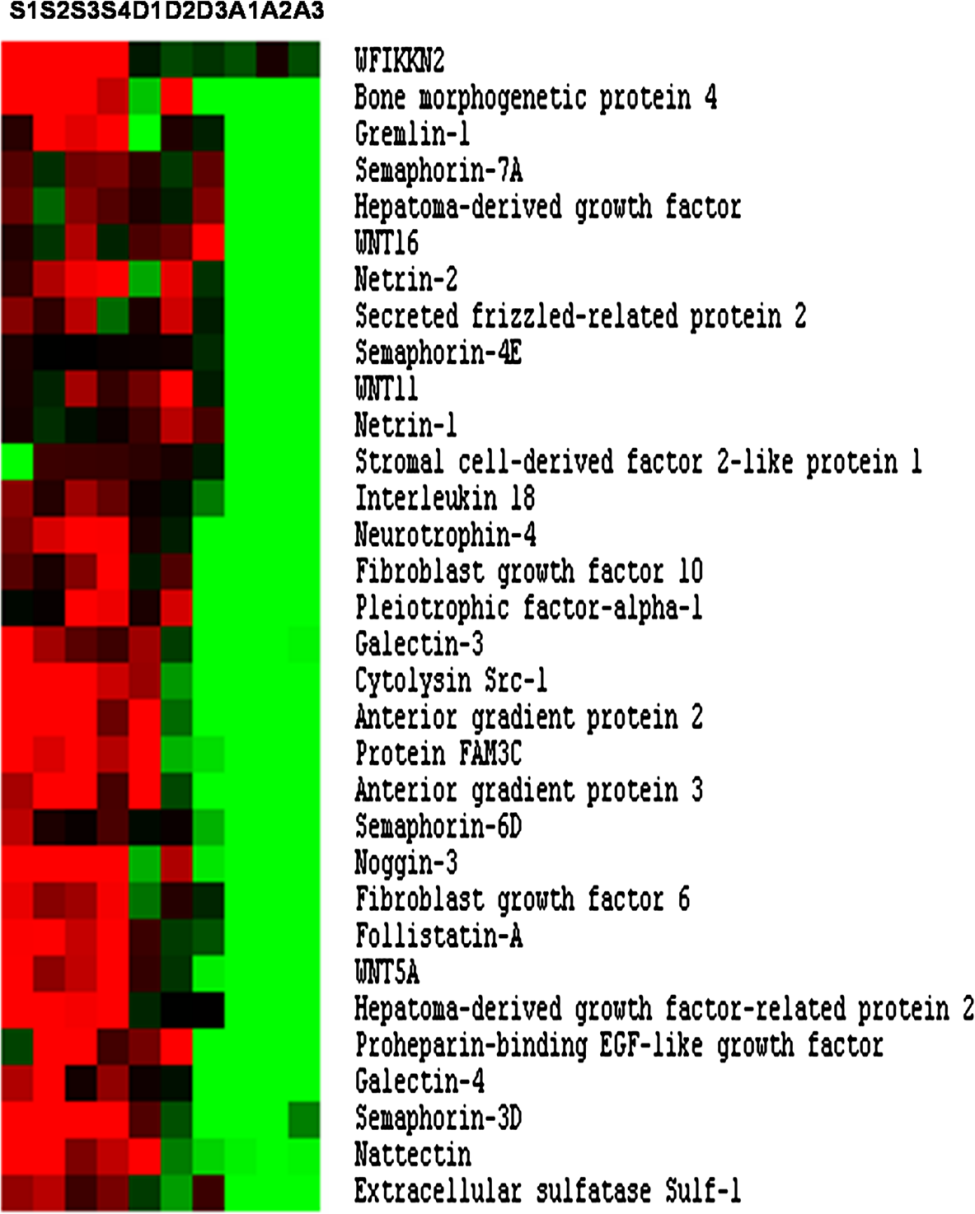
**Supervised clustering of secreted factors overexpressed in hyperplastic areas.** Columns are as in Figure 2.

When compared to adult fast muscle, superficial hyperplastic areas of the late embryo myotome were found to up-regulate a large set of secreted factors that are presented in Figure 6. Some of these factors, such as BMP4 and Wnt proteins (i.e., Wnt5a, Wnt 11 and Wnt 16), are morphogens known to regulate developmental processes. Secreted antagonists of BMP (Noggin3 and Gremlin-1), Wnt (sfrp2) and myostatin (follistatin and Wap, kazal, immunoglobulin, kunitz and NTR domain-containing protein 2 (WFIKKN2)) were also found to be up-regulated. Growth factors overexpressed in hyperplastic zones included FGF10, FGF6, IL18, Hepatoma derived growth factor, Hepatoma derived growth factor-related protein 2, anterior gradient proteins 2 and 3, Neurotrophin 4, pleiotrophic factors alpha-1 and proheparin-binding EGF-like growth factor. Other factors overexpressed in the late embryonic myotome included Nattectin, Netrin 1, Netrin 2 and galectin 3 and 4, which regulate cell-cell or cell-extracellular matrix interactions, cytolysin Src-1, which is involved in membrane reorganisation, Fam3C, and several members of the semaphorin family, such as semaphorin 3D and 7A. Finally, we observed the up-regulation of sulf-1, an extracellular endosulfatase that regulates growth factor signalling for satellite cell differentiation.

### In situ hybridisation of transcripts up-regulated in laser-captured peripheral hyperplastic domains of the myotome

**Figure 7:**
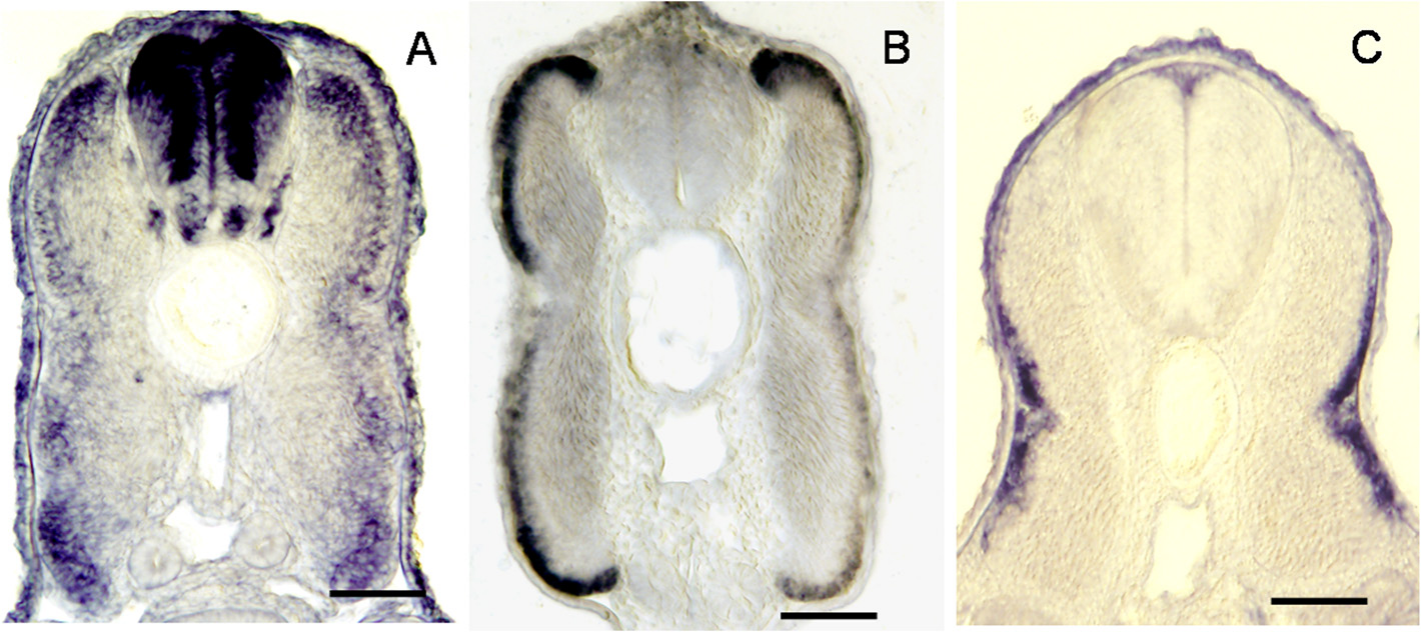
**Examples of in situ hybridisation of transcripts found to be up-regulated in laser- captured hyperplastic areas.** Transverse sections through the trunk of a 17-day-old trout embryo. Hes6 transcript accumulates in growth zones (**A**), BMP receptor type-1B transcript is mainly detected in dorsal and ventral lips of the dermomyotome (**B**) and gremlin transcript accumulates in the central domain of the dermomyotome (**C**). Horizontal line, 60 μm.

One hundred fifty genes found to be up-regulated in laser-captured superficial hyperplastic areas *versus* adult fast muscle and representing a broad range of biological functions were selected and their expression was investigated by whole-mount in situ hybridisation on 17-day-old prehatching trout embryos. At this stage, the dermomyotome-like epithelium, which provides myogenic cell precursors for lateral fast muscle expansion, is still apparent at the surface of the myotome [5]. Approximately 15% of the clones tested gave a clear signal within the myotome of the late trout embryo indicating that a large fraction of the transcripts up-regulated in hyperplastic zones are expressed at a level too low to be detected in situ using our hybridisation conditions. When present, signals were always observed at the surface of the myotome, either in the myogenic progenitors forming the dermomyotome-like epithelium, as in the case with gremlin, SFRP2, brother of CDO (BOC) and Kin of Irre 3 or more medially, in the differentiating myofibres, as observed for Hes-6, M-cadherin and c-myc (Figure 7 and Additional file 4).

## Discussion

By taking advantage of laser capture microdissection and microarray technologies we aimed in this study to discover genes that potentially regulate myotube neoformation in fish. Combining these experimental approaches we identified nearly 7000 genes that were up-regulated in superficial growth (hyperplastic) zones of the late trout embryo myotome compared to adult myotomal muscle. In these zones, our transcriptomic analysis revealed the up-regulation of canonical genes known to have a role in controlling myogenesis, such as Pax3, Pax7 and the four bHLH myogenic factors. This observation indicates that muscle hyperplasia depends on the genetic regulatory pathways that regulate the initial (embryonic) myogenesis. The overexpression of the muscle progenitor markers Pax3 and Pax7 is likely the result of undifferentiated dermomyotome-derived myogenic cells, which are transiently stockpiled in the lateral fast muscle before they differentiate into new myofibers (Steinbacher et al., 2008), in the captured material. Along with MRF genes, we observed the up-regulation of Tsh3, ARX, meis1 and the homeodomain containing protein pbx1, all of which have been reported to modulate the activity of MRF during skeletal muscle differentiation [19-22]. Interestingly, we observed the overexpression of a variety of transcriptional regulators for which a function in myogenesis has not been shown or is poorly documented; for example, we noted the up-regulation of seven distinct members of the Myc family. Using in situ hybridisation, we further showed that c-myc transcript is detectable in differentiating myotubes indicating that c-Myc not only regulates cell growth, apoptosis and metabolism as classically reported [23], but may also activate, at least in myogenic cells, the differentiation machinery. Among the Hes gene family members overexpressed in hyperplastic zones, we found Her6, hey1 and hey2 which are transcribed in the developing primary myotome of zebrafish [24]. In addition, we showed in this study that Hes6 and hairy related-9 transcripts are detectable in superficial hyperplastic zones using in situ hybridisation. In keeping with a possible role for Hes6 in trout muscle hyperplasia, it is worth noting that the microinjection of Hes6 RNA into *Xenopus* embryos induces an impairment of terminal differentiation and an expansion of the myotome [25]. Sox5 and Sox11 transcripts were also up-regulated in laser-captured samples from the peripheral domain of the myotome. We have recently shown that Sox5 is expressed in myogenic cells from the dermomyotome-like epithelium notably at the level of the dorsal and ventral lips [26]. We report in this study a similar pattern of sox11 expression, indicating that these two genes are transcribed in dermomyotome-derived cells prior their differentiation. In addition to DNA-binding transcriptional regulators, we observed the up-regulation in hyperplasic area of a large set of genes involved in chromatin remodelling. Specifically, we observed the up-regulation of Ezh2, an essential component of the polycomb-repressive complex, which has been recently reported to control self-renewal and preservation of the transcriptional identity of skeletal muscle stem cells [27]. Additionally, we report the overexpression of histone modifying enzymes including protein arginine methyltransferase 4 (PRTM4/CARM1) and 5 (PRTM5), which, in zebrafish, have a major role in controlling MRF expression and proper myogenesis [28]. Among the SWI/SNF chromatin remodelling enzymes overexpressed in our analysis it is worth noting that Brg1/Smarca4 has been shown to alter chromatin structure at myogenic loci facilitating transcription [29].

Myoblast fusion is required for muscle fibre formation. This step depends on cell-cell contact that is mediated by proteins present at the surface of the myoblats [30] (Krauss et al., 2005). Among the large set of membrane proteins up-regulated in hyperplastic zones were found many genes encoding Ig-domain containing transmembrane proteins such as Kin of irre 3, Ncam, N-cadherin, M-cadherin and Brother of CDO, all of which are known to influence cell-cell interactions during myoblast differentiation or fusion [30]. Surprisingly, in contrast to M-cadherin, which is expressed in differentiating myoblasts located at the periphery of the expanding myotome [31], Kin of Irre 3 and brother of CDO were found to be transcribed primarily in the external dermomyotome-like epithelium, similar to what is observed for N-cadherin [31]. This indicates that the transcription of Kin of Irre 3 and brother of CDO takes place early in the myogenic progenitor cells that participate in the second wave of myofibre formation and does not depend on MRF activity. Interestingly, we found that Junctional adhesion molecule Jam2b which is closely related to jam2a (Jam-B) and Jam3b (JAM-C), both critical for myocyte fusion [32], was also up-regulated along with protogenin which is closely related to the promyogenic transmembrane protein Neogenin [30]. The Ig superfamily members CD 166 and CD 276, which were up-regulated in our microarray, have also been detected at the surface of mouse C2C12 myoblasts [33], suggesting a role for these two proteins in early myoblast-myoblast interactions. Other membrane proteins that were both up-regulated in superficial hyperplastic zones in our study and that have been detected at the surface of C2C12 cells included cleft lip and palate transmembrane protein1, trophoblast glycoprotein, tissue factor/CD142, Ephrin type A receptor 2, tetraspanin 3 and 4 and fibroblast growth factor 4 (FGFR4) [33]. FGFR4 is highly expressed during chick embryo muscle differentiation [34] (Marics et al., 2002) and muscle regeneration [35]. Our data further support the involvement of FGFR4 in fish muscle hyperplasia as FGF6, a secreted ligand that binds to FGFR4 [36], is overexpressed in laser-captured hyperplastic zones. Other secreted factors that may act in an autocrine and/or paracrine manner to regulate muscle hyperplasia included anteriorgradient protein 2 which acts as a growth factor for blastema cells during regeneration of salamander limbs [37], and Neurotrophin 4, which acts as a regulator of the development, maintenance and regeneration of skeletal muscle fibres [38]. In addition, the expression of both Hepatoma-derived growth factor and Hepatoma-derived growth factor-related protein 2 was up-regulated in hyperplastic zones. Hepatoma derived growth factor is a heparin binding protein that promotes the proliferation, differentiation and migration of various cell types, such as vascular smooth muscle cells [39].

The Sema3D and sema7A proteins that were up-regulated in our microarray, are also produced by C2C12 differentiating myogenic cells [40]. These secreted proteins were initially identified as regulators of axon guidance [41] and, subsequently, were shown to participate in myogenic differentiation [42,43]. Superficial growth zones exhibits high levels of expression of various morphogens or secreted antagonists of morphogens, suggesting that these areas are subjected to complex overlapping morphogenic signals. An example of this from our analysis is the up-regulation of both Gremlin-1 and SFRP2. Gremlin-1 is known to inhibit Tgfβ/BMP activity and thus favours myogenic cell differentiation [44]. However, SFRP2, by inhibiting the myogenic activity of Wnt, is predicted to prevent precocious myogenic differentiation. Interestingly, the expression of Wnt5 and Wnt11 is up-regulated in hyperplastic zones along with the transmembrane receptors frizzled 3 and 7, the protocadherin Fat1, the dishevelled interactor dact1 and the planar cell polarity effectors Vang-like1 and Vang-like2. This suggests that a pathway similar to planar cell polarity [45], which is notably involved in the oriented elongation of early muscle fibres [46], may regulate the formation of new muscle fibres at the surface of the trout primary myotome.

## Conclusions

In the present study, LCM and microarray analysis were used as tools to characterise the transcriptomal landscape of the superficial growth zones of the early fish myotome. Our data provide a valuable resource for the further characterisation of individual genes, sets of genes and signalling pathways that may control the neoformation of myotubes in fish. In addition, this work serves as a potentially useful source of information for the identification of novel genes that regulate muscle regeneration in vertebrates.

## Competing interests

The authors declare that they have no competing interests.

## Authors’ contributions

PYR coordinated the study, analysed the data and wrote the manuscript. JM performed microarray experiments and analysed the data. AF performed laser capture microdissection and RNA extractions of laser captured material. CR and VL performed in situ hybridisations. All authors read and approved the final manuscript.

## Supplementary Material

Additional file 1 Biological functions associated with hyperplasia-correlated genes as defined by Ingenuity Pathway Analysis. Click here for file

Additional file 2 List of the transcriptional regulators overexpressed in the superficial growth zones. Click here for file

Additional file 3 List of the membrane-associated proteins overexpressed in the superficial growth zones. Click here for file

Additional file 4 **In situ hybridisation of transcripts up-regulated in laser captured hyperplastic area.** Transverse sections through the trunk of a 17-day-old trout embryo. (A) Myc, (B) Meis 3, (C) Sox11, (D) AATF, (E) Hairy related-9, (F) Meis 1, (G) Kirrel3, (H) Brother of CDO (BOC), (I) Wnt16, (J) M-cadherin, (K) RCAS1, (L) Tetraspanin 13, (M) Vang-like 2, (N) Lin-28, (O) SFRP2, (P) Dapper 1. Click here for file
