# Supplementary figures and images for: Gene expression profiling of the hyperplastic growth zones of the late trout embryo myotome using laser capture microdissection and microarray analysis
Source: BMC Genomics. 2013 Mar 14;14:173. doi: 10.1186/1471-2164-14-173 (PMC3608082; doi:10.1186/1471-2164-14-173)

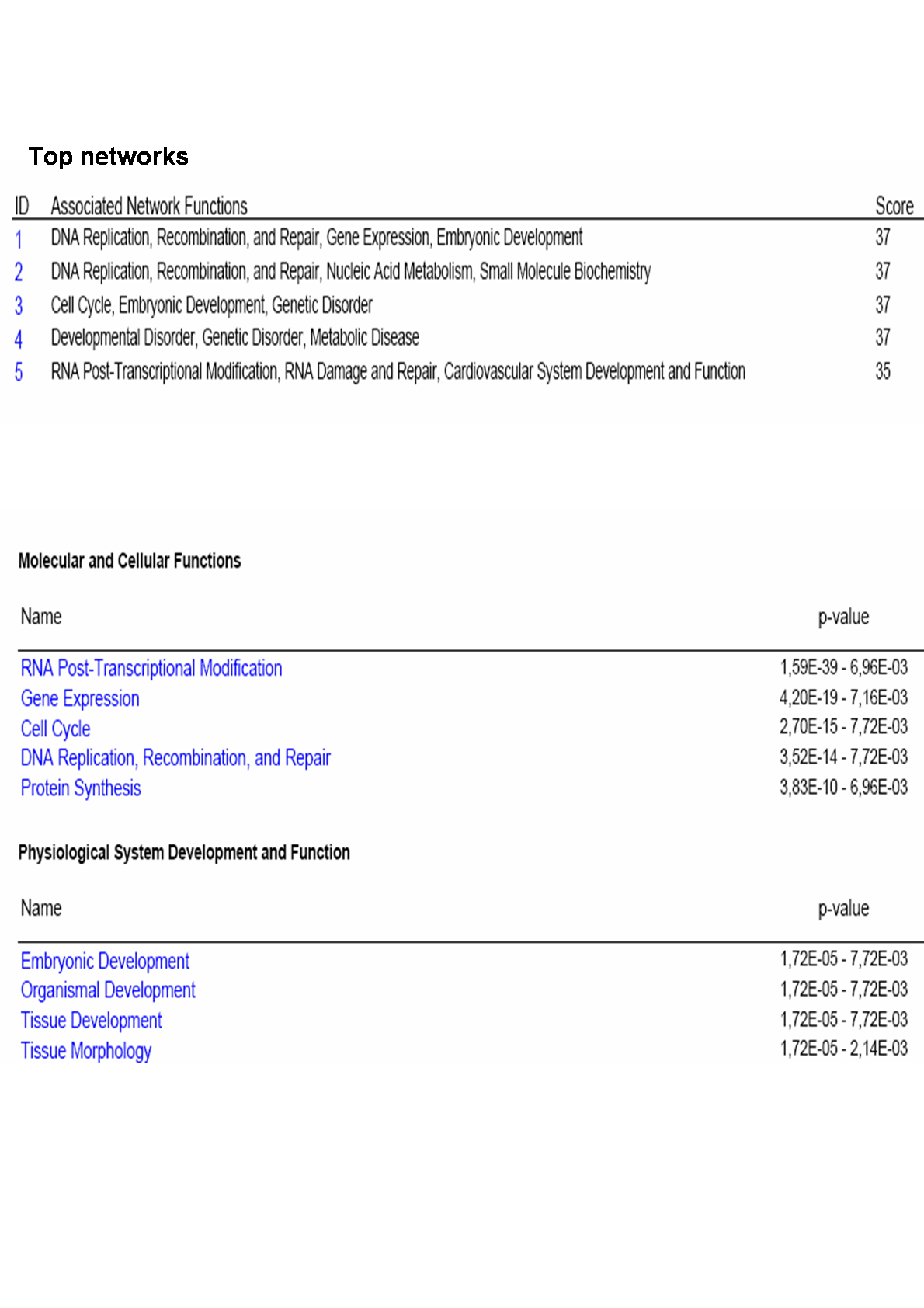

Supplement: Additional file 1 — Biological functions associated with hyperplasia-correlated genes as defined by Ingenuity Pathway Analysis. [file 1471-2164-14-173-S1.tiff]

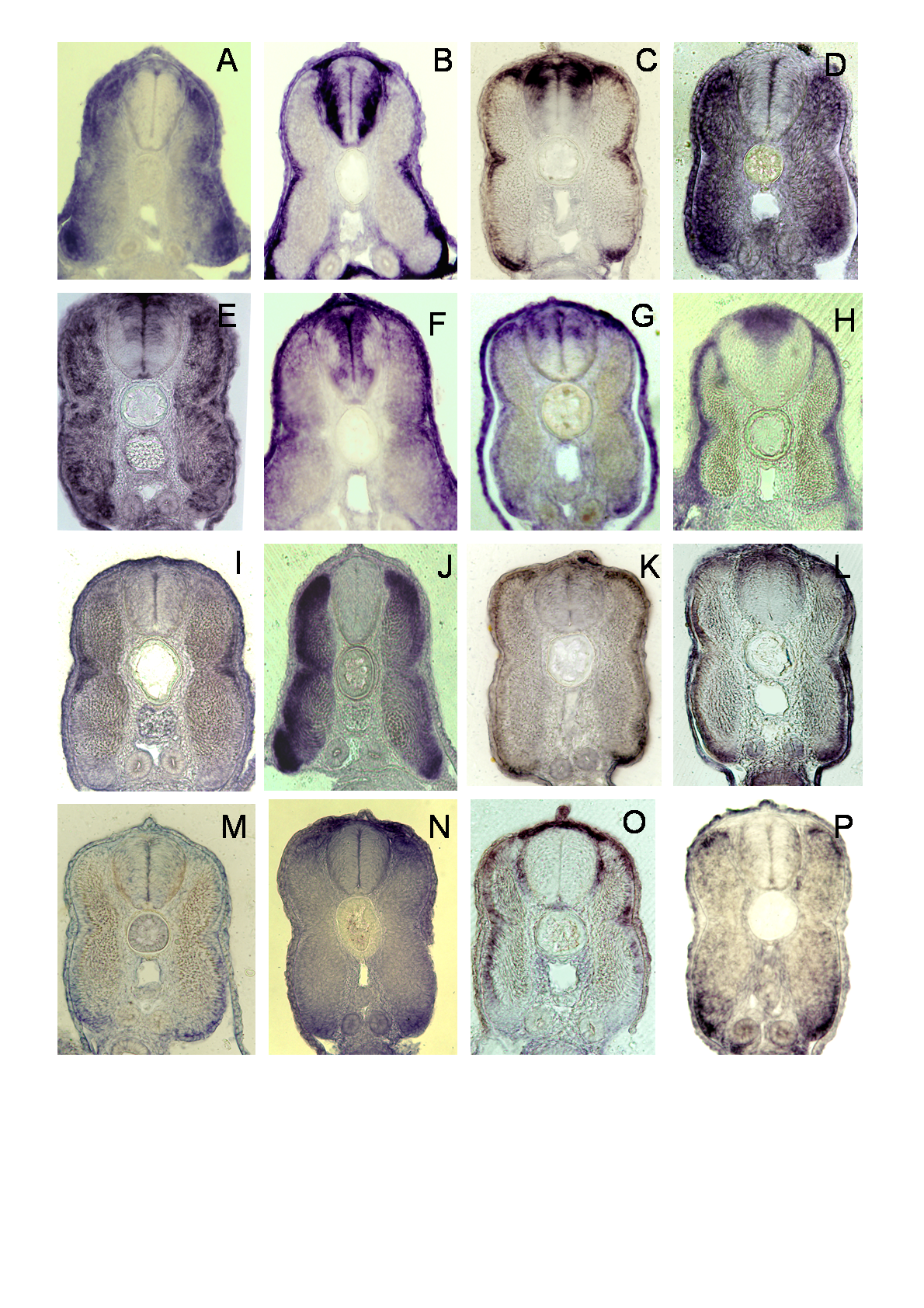

Supplement: Additional file 4 — In situ hybridisation of transcripts up-regulated in laser captured hyperplastic area. Transverse sections through the trunk of a 17-day-old trout embryo. (A) Myc, (B) Meis 3, (C) Sox11, (D) AATF, (E) Hairy related-9, (F) Meis 1, (G) Kirrel3, (H) Brother of CDO (BOC), (I) Wnt16, (J) M-cadherin, (K) RCAS1, (L) Tetraspanin 13, (M) Vang-like 2, (N) Lin-28, (O) SFRP2, (P) Dapper 1. [file 1471-2164-14-173-S4.tiff]
